# On the Interplay of Telomeres, Nevi and the Risk of Melanoma

**DOI:** 10.1371/journal.pone.0052466

**Published:** 2012-12-27

**Authors:** Clara Bodelon, Ruth M. Pfeiffer, Valentina Bollati, Julien Debbache, Donato Calista, Paola Ghiorzo, Maria Concetta Fargnoli, Giovanna Bianchi-Scarra, Ketty Peris, Mirjam Hoxha, Amy Hutchinson, Laurie Burdette, Laura Burke, Shenying Fang, Margaret A. Tucker, Alisa M. Goldstein, Jeffrey E. Lee, Qingyi Wei, Sharon A. Savage, Xiaohong R. Yang, Christopher Amos, Maria Teresa Landi

**Affiliations:** 1 Division of Cancer Epidemiology and Genetics, National Cancer Institute, Bethesda, Maryland, United States of America; 2 Center of Molecular and Genetic Epidemiology, Department of Environmental and Occupational Health, Università di Milano, Milan, Italy; 3 Fondazione Cà Granda, IRCCS Ospedale Maggiore Policlinico, Milan, Italy; 4 Cell and Developmental Biology Division, Universität Zürich, Zürich, Switzerland; 5 Department of Dermatology, M. Bufalini Hospital, Cesena, Italy; 6 Department of Internal Medicine, University of Genoa, Genoa, Italy; 7 Laboratory of Genetics of Rare Hereditary Cancers, IRCCS AOU San Martino-IST, Genoa, Italy; 8 Department of Dermatology, University of L’Aquila, L’Aquila, Italy; 9 Department of Surgical Oncology, The University of Texas, MD Anderson Cancer Center, Houston, Texas, United States of America; 10 Department of Epidemiology, The University of Texas, MD Anderson Cancer Center, Houston, Texas, United States of America; 11 Department of Community and Family Medicine, Center for Genomic Medicine, Dartmouth University, Lebanon, New Hampshire, United States of America; The Moffitt Cancer Center & Research Institute, United States of America

## Abstract

The relationship between telomeres, nevi and melanoma is complex. Shorter telomeres have been found to be associated with many cancers and with number of nevi, a known risk factor for melanoma. However, shorter telomeres have also been found to decrease melanoma risk. We performed a systematic analysis of telomere-related genes and tagSNPs within these genes, in relation to the risk of melanoma, dysplastic nevi, and nevus count combining data from four studies conducted in Italy. In addition, we examined whether telomere length measured in peripheral blood leukocytes is related to the risk of melanoma, dysplastic nevi, number of nevi, or telomere-related SNPs. A total of 796 cases and 770 controls were genotyped for 517 SNPs in 39 telomere-related genes genotyped with a custom-made array. Replication of the top SNPs was conducted in two American populations consisting of 488 subjects from 53 melanoma-prone families and 1,086 cases and 1,024 controls from a case-control study. We estimated odds ratios for associations with SNPs and combined SNP P-values to compute gene region-specific, functional group-specific, and overall P-value using an adaptive rank-truncated product algorithm. In the Mediterranean population, we found suggestive evidence that RECQL4, a gene involved in genome stability, RTEL1, a gene regulating telomere elongation, and TERF2, a gene implicated in the protection of telomeres, were associated with melanoma, the presence of dysplastic nevi and number of nevi, respectively. However, these associations were not found in the American samples, suggesting variable melanoma susceptibility for these genes across populations or chance findings in our discovery sample. Larger studies across different populations are necessary to clarify these associations.

## Introduction

Telomeres are chromatin structures located at the ends of chromosomes, comprised of repeats of (TTAGGG)_n_ and telomere-binding proteins [1]. During DNA replication, the nucleotide repeats are lost, making telomeres progressively shorter. When a critical length is reached, cells enter into replicative senescence or apoptosis. If the cell can bypass the cell-cycle checkpoint with the ability to continue dividing, chromosomal instability and/or cancer can occur [1].

Melanocytic nevi (benign and dysplastic) are strong risk factors for cutaneous melanoma [2]–[4]. Benign melanocytic nevi are transient proliferations of melanocytes. After a replication phase, melanocytes within nevi seem to enter into replicative senescence and rarely experience apoptosis [5]. It is thought that they remain in this state for decades and only a few progress to melanoma for reasons that are not well understood [3]. It is possible that shorter telomere length in melanocytes within nevi may trigger the entry into senescence by limiting cell proliferation. This could in turn affect the capacity for malignant transformation or proliferation of malignant clones within nevi and thus the risk of melanoma. In fact, two prospective studies have reported that shorter telomeres were associated with a decreased risk of melanoma [6], [7]. However, telomere length has been found to be positively correlated with the number and size of nevi in a cross-sectional study in the UK [8].

Telomere length regulation is a highly complex process requiring numerous proteins. One previous study assessed the relationship between genetic variants in five telomere genes and the risk of melanoma and found two single nucleotide polymorphisms (SNPs) in TERT and one in TRF1 to significantly increase the risk of melanoma [9]. However, only five genes related to telomere maintenance were included in that study, but more than 30 genes are involved in the telomere biology. Furthermore, the study was limited by the relatively small number of melanoma cases (N = 218).

In the current study, we combined data from three case-control studies and one family study conducted in Italy, a Mediterranean population generally characterized by a wide range of pigmentation characteristics, intense sun exposure and lower incidence rates of melanoma in comparison to those found in the US and Australia [10]. We examined the associations of 39 genes related to the biology of telomeres, and 475 SNPs within these genes, with the risk of melanoma. We evaluated the top findings in two different American populations. In addition, we explored whether these genes were related to the presence of dysplastic nevi (defined as 5 mm or larger, predominantly flat, and with at least two of the following characteristics: variable pigmentation, indistinct borders, and an irregular outline [11], [12]) and to nevus count in control subjects. We also explored whether number of nevi or presence of dysplastic nevi interact with SNPs in telomere biology genes, to assess whether genetic susceptibility has a role in the progression between nevi and melanoma. Finally, we tested for the association between telomere length and the risk of melanoma, nevus count, presence of dysplastic nevi and SNPs within telomere-related genes.

## Methods

### Ethics Statement

The study was approved by the Bufalini Hospital, the University of L’Aquila, the Genoa San Martino Hospital and the National Cancer Institute Ethics Committees and written informed consent was obtained from all participants.

### Study Populations

Subjects from three case-control studies of sporadic melanoma and one study of familial melanoma conducted in Italy were analyzed. The methods for the first case-control study (CCS1) have been previously described [13], [14]. Briefly, cases were incident sporadic melanoma patients, without a family history of melanoma, aged 17–77, negative for the CDKN2A/CDK4 mutations, diagnosed between December 1994 and January 1999 at the Dermatology Unit of Maurizio Bufalini Hospital in Cesena, a referral point for the Emilia-Romagna and Northern Marche regions in Northern Italy. During the study period, the Bufalini Hospital examined approximately 85% of all melanoma patients diagnosed in the area, as verified by comparison of patient lists with the Romagna region cancer registry and with records of melanoma diagnoses from the main hospitals of the area. Controls were spouses or close friends of the cases, patients treated at the same hospital for minor accidental trauma or healthy hospital personnel recruited during the same period without a history of melanoma and coming from the same geographical areas as the cases. Controls were frequency-matched by age and gender to cases. A single dermatologist (D.C.) performed skin examinations for all study subjects to determine pigmentation characteristics, freckles, nevus count (on the back only), presence of dysplastic nevi, as well as other skin traits. Questionnaires were administrated to participants to obtain information on demographic characteristics, family and medical history and sun exposure behaviors. A total of 227 cases and 173 controls with blood samples were included in the present study.

The methods for the second case-control study (CCS2) have also been published [15]. Briefly, sporadic melanoma patients (not tested for the CDKN2A/CDK4 mutations), diagnosed at the Departments of Dermatology of the Universities of L’Aquila, Florence or Modena in central Italy, aged 17–82, were recruited between 2000 and 2002. Subjects treated for diseases unrelated to melanoma at the Surgery and Internal Medicine Departments of the corresponding Universities were recruited as controls for the study. They were frequency matched by sex and age (±1 year) and study area. Information on demographic characteristics, family and medical history, sun exposure, and pigmentation nevi was collected using a standardized questionnaire. Clinical examination of all subjects was performed by two dermatologists (K.P. and M.C.F.). A total of 264 cases and 165 controls with blood samples were included in the present study.

The third case-control study (CCS3) was of sporadic melanoma cases diagnosed between 2000–2007 at the units of dermatology, medical oncology and plastic surgery of the National Cancer Research Institute and San Martino Hospital, Genoa, in Northern Italy [16]. Melanoma cases were older than 18 years of age, without family history of melanoma and negative for the CDKN2A/CDK4 mutations. Similarly, subjects without a history of melanoma, older than 18 years of age were recruited at the same hospital during the same period. A trained interviewer obtained information from study subjects on demographic characteristics, family and medical history, sun exposure, and pigmentation characteristics using a standardized questionnaire. All melanoma cases and 54.8% of the controls were also examined by experienced dermatologists or plastic surgeons that also filled out the questionnaire. The study was approved by the A total of 207 cases and 207 controls with blood samples were included in the present study.

Finally, the familial melanoma study (FS) consists of 62 families from Northern Italy with at least two relatives diagnosed with melanoma who were recruited at the Dermatology Unit of Maurizio Bufalini Hospital in Cesena [17]. Some of the melanoma cases were deceased at the time of recruitment and so blood samples were obtained from 96 melanoma patients and 225 unaffected individuals. The protocol for skin examination and questionnaire administration was identical to CCS1, although the questionnaires differed slightly.

### Gene and SNP Selection and Genotyping

Thirty-nine genes coding for proteins that are known to directly interact with telomeres or to regulate these proteins were selected for our study, following Mirabello *et al.* [18] (Table S1). TagSNPs in these genes and surrounding regions, comprising of 20kb upstream of the 5′ of each gene and 10kb downstream of the 3′ of each gene, were selected if they had a minor allele frequency (MAF) larger than 5% and pairwise correlation (r^2^) smaller than 0.80 in the HapMap2 Project database. In total, 747 SNPs were selected for genotyping using an iSelect Illumina Infinium custom array at the NCI Core Genotyping Facility [19]. Two hundred and thirty SNPs were excluded due to failing the assay (n = 93), being monoallelic (n = 1), having less than 90% completion (n = 93), having less than 95% concordance (n = 5), not possible to genotyped in the custom array (n = 20) or having Hardy-Weinberg equilibrium (HWE) less than 0.05/20,809 (n = 18), with 20,809 being the total number of non-failing SNPs in the iSelect Illumina Infinium array. A total of 517 SNPs were genotyped. Analyses were restricted to SNPs with MAF of at least 1% in controls, resulting in 476 SNPs.

### Replication Data Sets

Two datasets were used to verify whether the associations found in the Mediterranean population were also present in American subjects who differed by residence, sun exposure, pigmentation characteristics and family history of melanoma. The first was a family study comprised of 562 individuals (183 melanoma cases) from 53 melanoma-prone families (23 of which had CDKN2A germline mutations) with at least two living first degree relatives diagnosed with invasive melanoma and ascertained through health care professionals or self-referrals. Two adult controls were selected for each case, including blood-related or unrelated family members. The study was approved by the National Cancer Institute Clinical Center Institutional Review Board and all subjects gave informed consent. Genotyping was conducted using the same iSelect Illumina Infinium custom array at the NCI Core Genotyping Facility.

The second study was a hospital-based case-control study of non-Hispanic white subjects recruited at the MD Anderson Cancer Center [20]. Specifically, there were 1,804 melanoma cases and 1,026 cancer-free controls (friends or acquaintances of patients reporting to other clinics), who were frequency-matched on age and sex, completed a comprehensive skin and lifestyle questionnaire, and whose DNA passed quality control filters for genotyping for a genome wide-association study. Genotyping was conducted at the John Hopkins University Center for Inherited Disease Research (CIDR), using the Illumina HumanOmni1-Quad_v1-0_B array. Genotypes were called using the BeadStudio algorithm. MACH was used to impute ungenotyped SNPs according to 1000 Genomes Project data using the HapMap2 reference data. Information was not available on dysplastic nevi. The study was approved by the MD Anderson Institutional Review Board and all individuals provided informed consent.

### Relative Telomere Length Measurements

Information on telomere length was available for 368 subjects from CCS1 and FS (172 melanoma patients and 196 non-melanoma subjects). DNA was extracted from peripheral blood leukocytes. Telomere Length (TL) was measured using a quantitative real-time method described by Cawthon [21]. This method measures the relative TL in genomic DNA by determining the ratio of telomere repeat copy number (T) to a single copy gene (the human beta-globin gene, S) copy number (T/S ratio) in experimental samples relative to a reference sample. The two reactions were run in triplicate in two different plates for all samples. SYBR green dye was used to monitor DNA synthesis *versus* background fluorescence, as well as the melting temperature of the product. Each plate also contained a pool of DNA with serial 1∶2 dilutions to compute a standard curve (1.25 ng to 40 ng). This standard curve was generated from a serially diluted DNA pool (obtained from 20 DNA samples randomly selected from the samples tested in the present study), ranging from 1.25 to 40 ng in each plate, so that relative quantities of T and S (in nanograms) could be determined from it. The average of the three T measurements was divided by the average of the three S measurements to calculate the average T:S ratio or relative telomere length [21].

### Statistical Analysis

We conducted SNP-based, gene-based, functional group based and overall association analyses. All computations were done using the R (version 2.13.2) software. HWE was calculated among controls using an exact test [22] (Table S1). One of the SNP genotypes (rs7973425 in the DDX11 region) deviated from Hardy–Weinberg Equilibrium in the control study population after correcting for multiple testing and it was excluded from the analyses (Table S1). Linkage disequilibrium (LD) was estimated using the r^2^ measure among the genotyped SNPs, and the corresponding heatmap was computed using a modified version of the R statistical package LDheatmap [23].

#### SNP-based association analysis

For each SNP genotype separately, we computed an odds ratios (OR) and the corresponding standard errors (SE) for the association with risks of melanoma using unconditional logistic regression models for each of the case-control studies separately and conditional logistic regression (conditioning on family) for the family study, adjusting for age (continuous) and sex. We assumed an additive model in the number of minor alleles (i.e., SNP genotypes were coded as 0, 1, or 2) and fitted the genotype with a trend in the logistic models. If the number of subjects was less than 5 for one genotype, we used a dominant model. We then combined study-specific ORs using a random effects meta-analysis [24]. However, similar results were obtained using fixed effects models (data not shown). We further adjusted the OR for a “nevus score”, comprised of nevus count and presence of dysplastic nevi. These nevus scores were computed using factor analysis for those individuals who did not have missing values on the nevus count and presence of dysplastic nevi. Adjustment for the nevus score grouped into quartiles yielded similar OR estimates to those adjusted for only age and sex (Table S2). Therefore, we presented here only the results from models adjusting for age and sex. Analyses for the association of risk of dysplastic nevi (coded as absent or present) with SNPs were conducted in non-melanoma participants using the same approach as described above.

Poisson regression models adjusted for age and sex were used to explore the association between nevus count and the SNPs in controls from CCS1 and FS to compute incident rate ratios (IRR). We added a SNP genotype and age interaction to adjust for the changes of nevus count with age [8]. To account for model assumptions (and correlations among individuals in the FS), we used a robust sandwich variance estimator.

Finally, we explored whether the presence of dysplastic nevi modified the association between SNP genotypes and melanoma risk by adding an interaction term between dysplastic nevi and SNP genotypes in the SNP-based analysis. A similar analysis was conducted for nevus count.

We corrected for multiple testing using the Bonferroni method, which is likely conservative, because of the correlation across SNPs. After correction, P-values needed to be lower than 1.05×10^−4^ for significance at the 5% level.

Analysis for the replication data sets were conducted as described above, except for nevus count. In the family study, the variable was collected as an ordinal variable with four categories and analyzed using a multinomial model. In the hospital-based study, nevus count was coded as a binary variable.

#### Gene-based, group-based and overall association analysis

SNP-based P-values within a gene region were combined by means of an adaptive rank truncated product (ARTP) approach to compute the gene-based P-value [25]. In this approach, SNP-based P-values within a gene region are combined using a truncated product method, in which the truncated point is optimized among a set of candidates. This product defines a test statistic and the gene-based P-value is the result of this statistic. Significance of the gene-based P-value was assessed using a permutation procedure with 10,000 permutations. This approach allows for LD within a gene region and allows for flexibility in the number of SNPs to include in the P-value calculation. Nine SNPs mapped to two different gene regions each and were included in both gene analyses (rs4873772 and rs762679 to MCM4 and PRKDC; rs2700, rs3093942, rs4981158, rs10147163, rs7161611 and rs1713419 to PARP2 and TEP1). The thirty-nine genes were grouped in several functional groups [18] and functional group P-values were computed by combining the SNP-based P-values from the genes in the corresponding group using the ARTP algorithm. The overall P-value combined all the SNP-based P-values using the ARTP package.

#### Telomere length association analysis

Telomere length was transformed using natural logarithms. We computed associations between telomere length and different demographic and sun-related characteristics among non-melanoma subjects using linear regression, adjusted for age and study (CCS1 vs. FS). We chose to adjust for study instead of using meta-analytic estimates, due to the small number of unaffected subjects with telomere length, leading to unstable estimates in the study-specific analysis. The association between telomere length and the risk of melanoma was explored using unconditional (in CCS1) and conditional (in FS) logistic regressions adjusting for age and sex using telomere length as a continuous variable. Study-specific ORs were combined using a random effects meta-analysis [24].

## Results

A total of 796 melanoma cases and 770 unaffected subjects were included in our genetic analysis. Melanoma patients and non-melanoma participants were similar in age and sex (Table 1). Cases were more likely to have increased number of nevi, presence of dysplastic nevi and to report more sun exposure compared to unaffected individuals.

**Table 1:** Distribution of characteristics of study subjects.

Characteristics	Cases(N = 796) n (%)	Unaffected(N = 770) n (%)
*Age (years)*		
<30	93 (11.7)	106 (13.8)
30–39	142 (17.9)	147 (19.1)
40–49	152 (19.1)	161 (21.0)
50–59	174 (21.9)	144 (18.8)
≥60	234 (29.4)	210 (27.3)
*Sex*		
Males	377 (47.4)	381 (49.5)
Females	419 (52.6)	389 (50.5)
*Nevus count*		
<10	244 (32.8)	238 (36.4)
10–50	289 (38.9)	308 (47.1)
>50	210 (28.3)	108 (16.5)
*Dysplastic nevi*		
No	457 (62.4)	463 (74.8)
Yes	275 (37.6)	156 (25.2)
*Chronic sun exposure* *		
No	467 (67.3)	343 (72.8)
Yes	227 (32.7)	128 (27.2)
*Intermittent sun exposure*		
Very little	237 (30.0)	227 (32.5)
Some	299 (37.9)	313 (44.8)
High	253 (32.1)	158 (22.6)

Numbers might no add to totals due to missing values. Data might not add up to 100% because of rounding.

* Not measured in the family study.

### SNP-based Findings

The C allele of the SNP rs2721173, located in the gene region RecQ protein-like 4 (RECQL4), was associated with an increased risk of melanoma (OR = 1.35, 95% CI: 1.13, 1.62; P-trend = 1.13×10^−3^; Figures S1 and S2 and Tables S2 and S3). Presence of dysplastic nevi was most strongly associated with the G allele of the SNP rs6011002 (OR = 3.30, 95% CI: 1.64, 6.61; P-trend = 7.75×10^−4^; Figures S3 and S4 and Tables S4 and S5), which is located in the regulator of telomere elongation helicase 1 (RTEL1) gene region. After Bonferroni correction, nevus count was significantly associated with the T allele of the SNP rs11955168 located in the RAD50 region (IRR = 2.79; 95% CI: 1.77, 4.38; P-trend = 8.68×10^−6^; Figure S5 and Table S6) and with the A allele of the SNP rs11850456 in the telomerase-associated protein 1 (TEP1) region (IRR = 0.41; 95% CI: 0.26, 0.64; P-trend = 1.04×10^−4^; Figure S5 and Table S7). Two SNPs, rs153045 and rs251796, in the TERF2 region, implicated in the protection of telomeres, that were previously found to be correlated with the number of nevi, were significantly associated with higher number of nevi in our study (IRR = 1.49; 95% CI: 1.2, 1.85; P-trend = 3.27×10^−4^ for rs153045; IRR = 1.32, 95% CI: 1.06, 1.66; P-trend = 0.01 for rs251796; Figures S5 and S6 and Tables S8, S9 and S10).

Analyses including interaction terms between SNP genotypes and the presence of dysplastic nevi or nevus count on the risk of melanoma yielded non-statistically significant results.

Our findings in the Italian study were not replicated in the two American datasets. In the US family study, which comprised 562 individuals (183 melanoma cases) from 53 melanoma-prone families, the C allele of rs2721173 in RECQL4 decreased the risk of melanoma by 17% (OR = 0.83, 95% CI: 0.36, 1.16; P-trend = 0.34), but did not reach statistical significance. In the hospital-based case-control study, comprised of 1,804 melanoma cases and 1,026 cancer free controls, the C allele of rs2721173 was not associated with melanoma risk (OR = 0.92, 95% CI: 0.79, 1.03; P-trend = 0.16). Similarly, SNP rs6011002 in RTEL1 was not associated with the presence of dysplastic nevi (OR = 0.65, 95% CI: 0.33, 1.29; P-trend = 0.22) in the family study (presence of dysplastic nevi was not available in the hospital-based case-control study). The SNP rs11955168 in RAD50 was not significantly associated with nevus count in the US family study (OR = 0.45, 95% CI: 0.10, 1.93; P-trend = 0.28) nor in the hospital-based study (OR = 1.37, 95% CI: 0.67, 2.83; P-trend = 0.39). Similarly, SNP rs11850456 in TEP1was not significantly associated with nevus count in the US family-study (OR = 0.76, 95% CI: 0.23, 2.54; P-trend = 0.66) nor in the hospital-based case-control study (OR = 0.77, 95% CI: 0.47, 1.29; P-trend = 0.32), although in the Italian and the two American studies it was associated with a reduced risk. The two SNPs that were previously reported to be significantly correlated with nevus count and that were significantly associated with number of nevi in our study were not associated in the two American studies, although the different categorization of nevus count and analysis techniques used in the different studies make difficult to directly compare them.

### Gene-based Findings

Similar to the SNP-based findings, the RECQL4 region had the strongest association with the risk of melanoma (gene-based P-value = 2.5×10^−3^; Table 2, Table S2 and Figure S2) and the RTEL1 region had the strongest association with risk of dysplastic nevi (gene-based P-value = 0.004; Table 3, Table S3 and Figure S4). The TERF2 region had the strongest association with nevus count (gene-based P-value = 0.009; Table 3, Table S4 and Figure S6).

**Table 2:** Gene-based association analysis with the risk of melanoma.

Gene	Number of SNPs in gene	P-value*
RECQL4	5	2.5×10^−3^
DDX1	10	0.03
TNKS2	5	0.06
RECQL5	4	0.12
PARP1	23	0.14
RAD51L3	13	0.14
TERF1	14	0.14
TEP1	37	0.16
RAD51C	6	0.17
NBN	17	0.18
RTEL1	13	0.22
RAD50	13	0.29
MRE11A	17	0.30
TERC	3	0.31
PIK3C3	3	0.33
WRN	16	0.33
NOLA1	8	0.39
TERF2	7	0.42
ATM	12	0.42
ACD	3	0.50
RAD54L	9	0.52
MYC	20	0.55
MCM4	2	0.58
MEN1	8	0.61
NOLA2	5	0.61
PINX1	29	0.62
NOLA3	14	0.65
BLM	28	0.69
TERF2IP	7	0.72
DDX11	4	0.72
PRKDC	7	0.72
TNKS	37	0.73
RAD51AP1	13	0.76
POT1	5	0.85
RECQL	21	0.86
TERT	12	0.87
PARP2	19	0.91
XRCC6	4	0.97
TINF2	9	0.98

* Based on a meta-analysis of the three case-control studies and one family study. Model was adjusted for age (continous) and sex. Genes ordered from most significant to least significant associations with outcome.

**Table 3:** Gene-based association analysis and risk of dysplastic nevi and number of nevi in unaffected subjects.

Dysplastic nevi			Nevus count		
Gene	Number of SNPs in gene	P-value*	Gene	Number of SNPs in gene	P-value**
RTEL1	12	4.2×10^−3^	TERF2	7	8.6×10^−3^
RAD54L	9	0.02	DDX1	10	0.01
PINX1	29	0.07	MEN1	8	0.04
NBN	17	0.10	TEP1	37	0.05
RECQL4	5	0.13	TERC	3	0.05
MYC	20	0.14	PARP2	19	0.05
RAD50	12	0.18	RAD50	14	0.10
RAD51AP1	13	0.21	RECQL4	5	0.21
PARP2	19	0.22	NOLA2	5	0.26
RAD51L3	13	0.22	MYC	20	0.31
NOLA2	5	0.24	RAD51L3	13	0.31
MEN1	7	0.28	RAD51AP1	13	0.36
TERC	3	0.32	RTEL1	13	0.39
TNKS	37	0.33	ATM	12	0.46
XRCC6	4	0.34	RAD51C	6	0.47
PRKDC	7	0.38	MCM4	2	0.50
DDX11	4	0.41	TNKS	37	0.50
MCM4	2	0.43	NOLA3	14	0.51
RECQL5	4	0.46	TNKS2	5	0.56
DDX1	10	0.46	TINF2	9	0.57
TEP1	37	0.48	ACD	3	0.60
RAD51C	6	0.48	WRN	16	0.60
TERF2	7	0.57	PINX1	29	0.60
NOLA1	8	0.60	XRCC6	4	0.63
POT1	5	0.62	DDX11	4	0.67
TNKS2	5	0.65	PARP1	23	0.67
BLM	26	0.65	PRKDC	7	0.74
ACD	3	0.65	TERF1	14	0.78
ATM	12	0.67	RECQL	21	0.79
TERF1	14	0.74	RAD54L	9	0.83
PARP1	23	0.84	PIK3C3	3	0.87
WRN	16	0.84	TERT	12	0.88
NOLA3	14	0.85	POT1	5	0.91
TERT	12	0.87	NOLA1	8	0.92
MRE11A	17	0.89	BLM	28	0.93
PIK3C3	3	0.93	MRE11A	17	0.94
RECQL	21	0.95	TERF2IP	7	0.96
TERF2IP	7	0.95	NBN	17	0.97
TINF2	9	0.99	RECQL5	4	0.98

* Based on a meta-analysis of the 3 case-control studies and the family study. Model was adjusted for age (continous) and sex.

** Based on a meta-analysis of case-control study and the family study using Poisson regression with robust variance. Model was adjusted for age (continous) and sex and interaction between age and SNP genotype. Genes ordered from the most to the least significant P-value.

### Functional Pathway-based Findings

We categorized the 39 genes into five different functional groups, as in Mirabello *et al*. [18] (Table S11). Similar to the gene-based results, the helicase subgroup, which included the RECQL4 region, was associated with melanoma risk (P = 0.03); a telomere related group, which included the RTEL1 region, was associated with dysplastic nevi (P = 0.11); and the shelterin subgroup, which included the TERF2 region, was associated with nevus count (P = 0.09).

In an analysis that combined p-values from all of the SNPs in the 39 telomere-related genes into a single summary p-value, we found no association with the risk of melanoma (overall P-value = 0.17), dysplastic nevi (overall P-value = 0.24) or nevus count (overall P-value = 0.17).

### Telomere Length

The telomere length analysis was based on 172 melanoma patients and 196 non-melanoma subjects from two studies (CCS1 and FS). A box-plot showing the distribution of telomere length among cases and unaffected individuals is available online (Figure S7). Among non-melanoma cases, telomere length was associated with age (P-trend: 4.72×10^−4^; Table 4), but was not associated with sex, nevus count, dysplastic nevi, or sun exposure. Furthermore, it was not significantly associated with the risk of melanoma (OR = 0.87; 95% CI: 0.46–1.64). In our study, telomere length was not associated with any of the selected SNPs among unaffected individuals (Figure S8).

**Table 4:** Relationship of telomere length with demographic and sun related characteristics in non-melanoma subjects.

Characteristics	Unaffected (N = 196) n (%)	Mean telomere length and 95% CI§	P-trend§
*Age (years)*			**4.72×10** ^−**4**^
<30	33 (16.9)	1.00 (ref.)	
30–39	43 (22.1)	0.94 (0.80, 1.12)	
40–49	43 (22.1)	0.89 (0.75, 1.06)	
50–59	43 (22.1)	0.87 (0.73, 1.03)	
≥60	33 (16.9)	**0.73 (0.61, 0.87)**	
*Sex*			0.85
Males	97 (49.5)	1.00 (ref.)	
Females	99 (50.5)	0.99 (0.89, 1.10)	
*Nevus count*			0.76
<10	26 (14.1)	1.00 (ref.)	
10–50	111 (60.3)	0.90 (0.77, 1.06)	
>50	47 (25.5)	0.99 (0.83, 1.19)	
*Dysplastic nevi*			0.85
No	120 (71.4)	1.00 (ref.)	
Yes	48 (28.6)	1.01 (0.88, 1.16)	
*Chronic sun exposure* *			0.13
No	90 (55.6)	1.00 (ref.)	
Yes	72 (44.4)	0.92 (0.82, 1.03)	
*Intermittent sun exposure*			0.57
Very little	57 (29.1)	1.00 (ref.)	
Some	92 (46.9)	0.95 (0.84, 1.08)	
High	47 (24.0)	0.96 (0.83, 1.11)	

Data might not add up to 100% because of rounding.

§ Compute only among unaffected individuals. Mean is the geometric mean. Adjusted by age and study (CCS1 vs. FS).

* Not measured in the family study.

## Discussion

In the current study, we combined data from three case-control studies and one family study conducted in Italy, and examined the associations of 39 genes related to the biology of telomeres, and 475 SNPs within these genes, with the risk of melanoma, presence of dysplastic nevi, number of nevi and telomere length.

The gene regions around RECQL4, RTEL1 and TERF2 were associated with melanoma, the presence of dysplastic nevi and number of nevi, respectively, in the Italian combined study. However, we did not find the same associations in two American studies with different study designs.

Previously, among the telomere-related genes, two SNPs in TERT, rs2853676 and rs2242652, were reported to have a significant association with melanoma in a Caucasian population [9]. However, these SNPs were not associated with melanoma in our population (OR = 1.17; 95% CI: 0.84, 1.63 for rs2853676; OR = 0.96; 95% CI: 0.66, 1.39 for rs2242652). Furthermore, the same study also reported two SNPs in TERF2 (rs153045 and rs251796) that were significantly correlated with number of nevi, but they did not report the size of the association after adjustment for confounding variables. We found that these two SNPs were significantly associated with higher number of nevi in our study. In the gene-based analysis, the TERF2 region had the strongest association with nevus count, suggesting that further research into this gene region and its role in nevi development may prove informative.

Shorter telomere length measured in prediagnostic blood samples was previously found to be correlated with number of nevi (P = 0.002) and associated with a reduced risk of melanoma, although not significantly (P-trend = 0.09) [7]. In a recent combined analysis of 3 prospective studies, shorter telomere length was associated with a reduced risk of melanoma (P-trend = 0.0003) [6]. In our study, shorter telomere length was not associated with number of nevi, but similar to the previous two analyses, telomere length was associated with a decreased risk of melanoma, although it did not reach statistical significance. Measurements of telomere length in our study were performed in samples collected retrospectively. However, this is unlikely to play an important role in our findings since one would have expected to observe a positive association with the risk of melanoma and we did not observe such an association.

This is the most comprehensive study of telomere-related genes and melanoma risk to date. In addition, we explored the associations between telomere length and nevi (dysplastic nevi and number of nevi). Further, we used gene-set and pathway approaches that allowed us to maximize our ability to detect effects that were only significant at the aggregate level, and not at the SNP level. We combined both case-control and family studies and assessed multiple variants in detail. However, the assessment of different characteristics, such as the number of nevi, could vary across studies and could have affected our measurements. For this reason, we performed analyses regarding number of nevi in the two studies (CCS1 and FS) in which skin examinations were performed by the same doctor. Moreover, we only considered the variable of presence/absence of dysplastic nevi instead of their number. Furthermore, we accounted for the potential heterogeneity in the studies by computing meta-analytic estimates, instead of pooling the data together. It is known that the number of nevi changes through the lifetime of an individual, with nevi count reaching its maximum around 35–40 years of age [8]. This could result in misclassification, leading to attenuated results. However, we included an interaction term between the number of nevi and age in our models to account for this effect modification by age.

Our findings may suggest a role for telomere-related genes in melanoma risk as observed for other cancers [26]–[29] and add insights into the complex biology of telomeres in melanoma development. However, despite the strengths of our study, our results were not confirmed in the two US populations, including a melanoma family study and a hospital-based case-control study. Differences in the ascertainment of nevi in the replication studies and related analyses could partially explain these results. Mediterranean populations generally differ from American and Australian populations, which experience a higher incidence of melanoma, in terms of pigmentation characteristics, intensity of sun exposure and genetic susceptibility [30]. These factors may also explain some discrepancies, but as in any epidemiological investigation, chance findings cannot be completely ruled out. Larger studies are needed to explore the role of these factors in relation to telomere genes and melanoma risk.

In summary, our results suggest that telomere-related genes might be related to the susceptibility of melanoma, dysplastic nevi and nevus count. Additional studies across different populations are warranted.

## Supporting Information

Figure S1 (DOC)

Figure S2 (DOC)

Figure S3 (DOC)

Figure S4 (DOC)

Figure S5 (DOC)

Figure S6 (DOC)

Figure S7 (DOC)

Figure S8 (DOC)

Table S1 (DOC)

Table S2 (DOC)

Table S3 (DOC)

Table S4 (DOC)

Table S5 (DOC)

Table S6 (DOC)

Table S7 (DOC)

Table S8 (DOC)

Table S9 (DOC)

Table S10 (DOC)

Table S11 (DOC)
