# Supplementary material for: On the Interplay of Telomeres, Nevi and the Risk of Melanoma
Source: PLoS One. 2012 Dec 27;7(12):e52466. doi: 10.1371/journal.pone.0052466 (PMC3531488; doi:10.1371/journal.pone.0052466)
Supplement: Figure S5 — (DOC) [file pone.0052466.s005.doc]

**Figure S5.** Association analysis between telomere biology SNPs and nevus count in non-melanoma subjects.

**
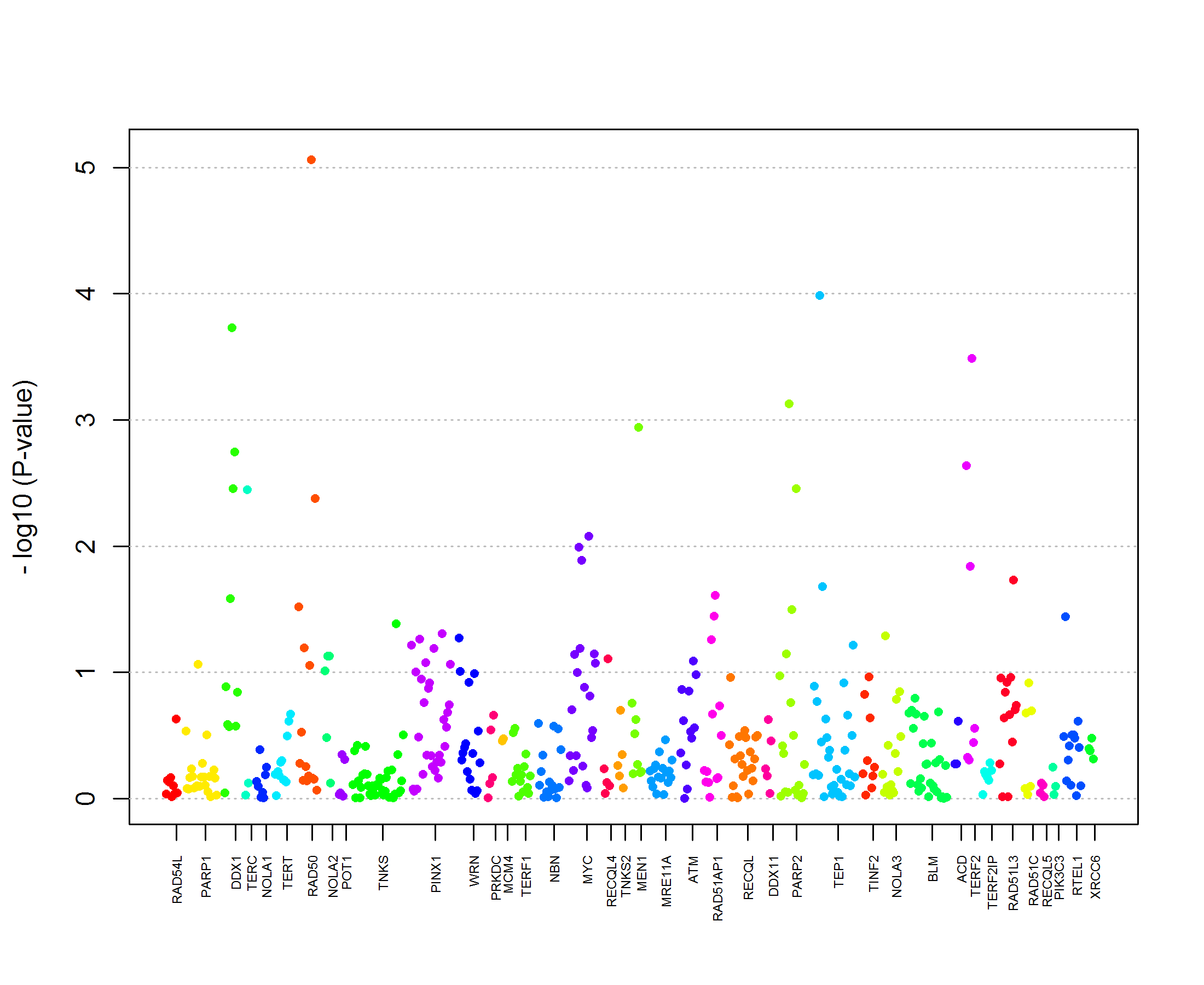
**

**Figure S5 legend.** Manhattan plot for the SNP-based association analysis and nevus count in non-melanoma subjects. Different colors correspond to different genes. Bonferroni correction at the 5% level corresponds to a P-value of 1.05×10^-4^ and the corresponding –log10(P-value) is 3.98.
