# Supplementary material for: On the Interplay of Telomeres, Nevi and the Risk of Melanoma
Source: PLoS One. 2012 Dec 27;7(12):e52466. doi: 10.1371/journal.pone.0052466 (PMC3531488; doi:10.1371/journal.pone.0052466)
Supplement: Figure S6 — (DOC) [file pone.0052466.s006.doc]

**Figure S6.** LD structure for the TERF2 region.

**
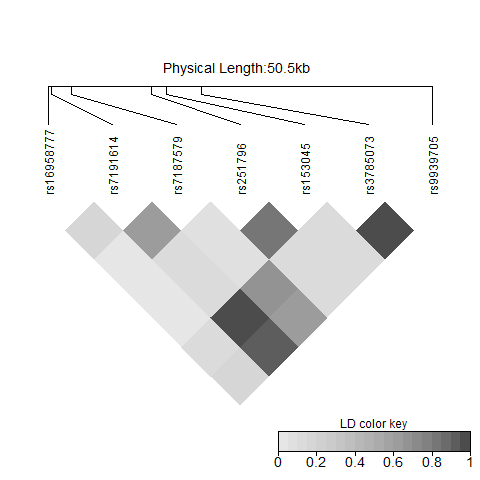
**

**Figure S6 legend.** LD structure for the SNPs in the TERF2 region in subjects without dysplastic nevi nor melanoma. Based on r^2^ values.
