# Supplementary figures and images for: On the Interplay of Telomeres, Nevi and the Risk of Melanoma
Source: PLoS One. 2012 Dec 27;7(12):e52466. doi: 10.1371/journal.pone.0052466 (PMC3531488; doi:10.1371/journal.pone.0052466)

**Figure S7.** Distribution of relative telomere length by disease status.

**
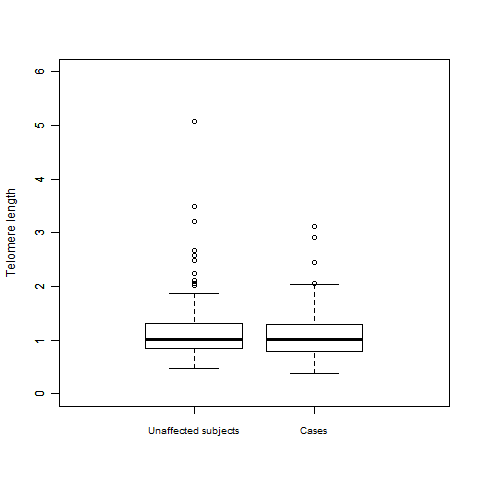
**

Supplement: Figure S7 — (DOC) [file pone.0052466.s007.doc]
