# Supplementary material for: On the Interplay of Telomeres, Nevi and the Risk of Melanoma
Source: PLoS One. 2012 Dec 27;7(12):e52466. doi: 10.1371/journal.pone.0052466 (PMC3531488; doi:10.1371/journal.pone.0052466)
Supplement: Table S1 — (DOC) [file pone.0052466.s009.doc]

**Table S1.** Gene and SNP information among melanoma controls.

| Gene | Chromosome | SNP | Common/Rare  alleles | Minor allele  frequency | Hardy-Weinberg  P-value |
| --- | --- | --- | --- | --- | --- |
| ACD | 16 | rs7200950 | C/T | 0.06 | 0.80 |
|  |  | rs6979 | G/A | 0.44 | 0.12 |
|  |  | rs7202185 | G/A | 0.04 | 0.65 |
| ATM | 11 | rs228606 | T/G | 0.48 | 0.45 |
|  |  | rs183459 | T/G | 0.34 | 0.21 |
|  |  | rs672655 | G/A | 0.34 | 0.23 |
|  |  | rs11212570 | G/A | 0.07 | 0.66 |
|  |  | rs228593 | G/A | 0.41 | 0.02 |
|  |  | rs1800057 | C/G | 0.04 | 0.34 |
|  |  | rs609261 | C/T | 0.34 | 0.59 |
|  |  | rs1801516 | G/A | 0.13 | 0.11 |
|  |  | rs595747 | T/C | 0.34 | 0.03 |
|  |  | rs227062 | A/G | 0.34 | 0.07 |
|  |  | rs227073 | C/G | 0.34 | 0.47 |
|  |  | rs17503908 | T/G | 0.11 | 0.88 |
| BLM | 15 | rs12441424 | G/A | 0.20 | 0.71 |
|  |  | rs12909306 | T/C | 0.13 | 0.20 |
|  |  | rs16944739 | C/T | 0.24 | 0.13 |
|  |  | rs7163604 | A/G | 0.23 | 0.04 |
|  |  | rs7184015 | G/T | 0.23 | 0.04 |
|  |  | rs8034371 | T/A | 0.13 | 0.89 |
|  |  | rs16944775 | G/A | 0.01 | 0.16 |
|  |  | rs7183841 | T/C | 0.13 | 1.00 |
|  |  | rs3784782 | C/G | 0.14 | 0.27 |
|  |  | rs2518967 | T/C | 0.30 | 0.77 |
|  |  | rs7170919 | C/T | 0.20 | 1.00 |
|  |  | rs16944798 | A/G | 0.02 | 0.29 |
|  |  | rs4932363 | G/A | 0.04 | 0.58 |
|  |  | rs8037430 | C/T | 0.23 | 0.03 |
|  |  | rs7175811 | G/A | 0.25 | 0.15 |
|  |  | rs16944840 | A/C | 0.02 | 0.38 |
|  |  | rs2270132 | A/C | 0.42 | 0.31 |
|  |  | rs389480 | G/A | 0.44 | 0.64 |
|  |  | rs2270131 | G/A | 0.15 | 0.64 |
|  |  | rs12592875 | T/C | 0.41 | 0.24 |
|  |  | rs16944863 | A/G | 0.07 | 0.15 |
|  |  | rs2532105 | G/A | 0.17 | 0.75 |
|  |  | rs414634 | C/A | 0.35 | 0.33 |
|  |  | rs439188 | C/T | 0.38 | 0.71 |
|  |  | rs8041787 | C/T | 0.22 | 0.24 |
|  |  | rs17183763 | G/C | 0.16 | 0.26 |
|  |  | rs441399 | A/G | 0.45 | 0.41 |
|  |  | rs3213196 | C/T | 0.16 | 0.27 |
| DDX1 | 2 | rs7560994 | A/C | 0.05 | 0.26 |
|  |  | rs7591019 | G/A | 0.28 | 0.03 |
|  |  | rs7601397 | A/G | 0.17 | 0.11 |
|  |  | rs16862784 | G/C | 0.12 | 0.58 |
|  |  | rs2890489 | A/G | 0.40 | 0.08 |
|  |  | rs3755130 | G/A | 0.43 | 0.12 |
|  |  | rs10169288 | C/G | 0.39 | 0.02 |
|  |  | rs2302928 | G/A | 0.27 | 0.07 |
|  |  | rs976016 | T/C | 0.14 | 0.58 |
|  |  | rs807629 | A/G | 0.37 | 0.38 |
| DDX11 | 12 | rs244496 | T/C | 0.08 | 0.06 |
|  |  | rs7973425 | C/G | 0.12 | 8.31×10^-8^ |
|  |  | rs11051239 | C/G | 0.39 | 0.50 |
|  |  | rs7957574 | G/C | 0.31 | 0.37 |
|  |  | rs4931434 | C/A | 0.24 | 0.37 |
| MCM4 | 8 | rs4873772 | G/A | 0.41 | 0.95 |
|  |  | rs762679 | A/T | 0.14 | 0.90 |
| MEN1 | 11 | rs677298 | A/G | 0.11 | 0.03 |
|  |  | rs11231879 | A/G | 0.06 | 0.77 |
|  |  | rs559566 | A/G | 0.45 | 0.86 |
|  |  | rs524386 | T/C | 0.09 | 0.03 |
|  |  | rs2957154 | T/C | 0.24 | 0.18 |
|  |  | rs4930680 | C/G | 0.02 | 1.00 |
|  |  | rs670358 | G/A | 0.08 | 0.27 |
|  |  | rs12790925 | G/A | 0.26 | 0.70 |
| MRE11A | 11 | rs10831224 | C/A | 0.09 | 0.50 |
|  |  | rs12270338 | C/A | 0.18 | 0.11 |
|  |  | rs581002 | C/T | 0.26 | 0.23 |
|  |  | rs10831228 | G/A | 0.12 | 0.69 |
|  |  | rs12708330 | C/A | 0.04 | 0.02 |
|  |  | rs588701 | G/A | 0.36 | 0.51 |
|  |  | rs592068 | T/C | 0.32 | 0.04 |
|  |  | rs569143 | G/C | 0.48 | 0.21 |
|  |  | rs496190 | A/G | 0.50 | 0.05 |
|  |  | rs7107508 | G/A | 0.04 | 0.01 |
|  |  | rs533984 | G/A | 0.43 | 0.28 |
|  |  | rs12708331 | C/T | 0.04 | 0.02 |
|  |  | rs610611 | T/A | 0.30 | 0.73 |
|  |  | rs13447623 | T/C | 0.22 | 1.00 |
|  |  | rs659349 | T/C | 0.44 | 0.25 |
|  |  | rs684507 | G/A | 0.50 | 0.07 |
|  |  | rs12708335 | G/T | 0.03 | 0.01 |
| MYC | 8 | rs16902346 | A/T | 0.04 | 0.72 |
|  |  | rs6470565 | C/T | 0.04 | 0.72 |
|  |  | rs6989598 | C/A | 0.04 | 0.72 |
|  |  | rs16902357 | C/T | 0.07 | 0.52 |
|  |  | rs10505505 | T/G | 0.05 | 0.76 |
|  |  | rs10108288 | C/T | 0.04 | 0.72 |
|  |  | rs16902359 | C/T | 0.11 | 0.78 |
|  |  | rs16902364 | A/C | 0.02 | 1.00 |
|  |  | rs17187428 | G/A | 0.08 | 0.07 |
|  |  | rs4645943 | C/T | 0.04 | 1.00 |
|  |  | rs4645946 | G/A | 0.02 | 1.00 |
|  |  | rs3891248 | T/A | 0.18 | 5.0×10^-4^ |
|  |  | rs4645956 | C/T | 0.13 | 0.34 |
|  |  | rs4645958 | C/G | 0.15 | 1.00 |
|  |  | rs10110283 | G/A | 0.02 | 0.27 |
|  |  | rs7003472 | C/G | 0.04 | 0.07 |
|  |  | rs12680047 | T/C | 0.38 | 0.67 |
|  |  | rs11782002 | T/C | 0.38 | 0.46 |
|  |  | rs4733550 | A/T | 0.47 | 0.08 |
|  |  | rs10956383 | T/C | 0.18 | 0.51 |
| NBN | 8 | rs2697677 | G/A | 0.13 | 0.02 |
|  |  | rs1881469 | A/T | 0.34 | 0.65 |
|  |  | rs13312971 | T/C | 0.01 | 1.00 |
|  |  | rs13312970 | G/C | 0.03 | 0.17 |
|  |  | rs2697679 | A/C | 0.34 | 0.79 |
|  |  | rs6998169 | T/A | 0.15 | 0.91 |
|  |  | rs12680687 | G/T | 0.26 | 0.33 |
|  |  | rs9297757 | C/A | 0.03 | 0.15 |
|  |  | rs16893166 | G/A | 0.01 | 1.00 |
|  |  | rs7830738 | C/T | 0.02 | 1.00 |
|  |  | rs6470522 | G/A | 0.13 | 0.53 |
|  |  | rs1805810 | A/G | 0.02 | 0.60 |
|  |  | rs1805812 | T/C | 0.06 | 0.77 |
|  |  | rs9649958 | T/G | 0.30 | 0.34 |
|  |  | rs1235369 | A/G | 0.05 | 1.00 |
|  |  | rs6985934 | C/T | 0.03 | 0.04 |
|  |  | rs1805796 | C/T | 0.34 | 0.65 |
| NOLA1 | 4 | rs7675460 | C/A | 0.38 | 0.01 |
|  |  | rs7671905 | C/T | 0.28 | 0.01 |
|  |  | rs6815517 | T/C | 0.29 | 0.01 |
|  |  | rs1002989 | T/C | 0.09 | 0.22 |
|  |  | rs11930711 | G/A | 0.08 | 0.14 |
|  |  | rs13140289 | A/T | 0.14 | 0.30 |
|  |  | rs10516559 | T/C | 0.12 | 0.31 |
|  |  | rs12508007 | C/T | 0.03 | 1.00 |
| NOLA2 | 5 | rs6601217 | A/G | 0.23 | 0.31 |
|  |  | rs6601221 | T/C | 0.44 | 0.13 |
|  |  | rs13189047 | C/A | 0.22 | 0.12 |
|  |  | rs7710186 | C/G | 0.29 | 0.19 |
|  |  | rs4976741 | T/G | 0.17 | 0.68 |
| NOLA3 | 15 | rs11631094 | G/T | 0.32 | 0.49 |
|  |  | rs2644243 | T/C | 0.38 | 0.21 |
|  |  | rs17817854 | T/C | 0.06 | 0.80 |
|  |  | rs6495662 | A/G | 0.22 | 0.41 |
|  |  | rs2169480 | A/G | 0.15 | 0.74 |
|  |  | rs8032347 | A/G | 0.14 | 1.00 |
|  |  | rs347797 | T/C | 0.45 | 0.41 |
|  |  | rs442873 | A/G | 0.05 | 0.56 |
|  |  | rs2279683 | C/A | 0.06 | 0.63 |
|  |  | rs2279685 | G/A | 0.06 | 0.06 |
|  |  | rs7162607 | G/A | 0.44 | 0.91 |
|  |  | rs4984236 | C/T | 0.40 | 1.00 |
|  |  | rs17236875 | T/C | 0.13 | 0.36 |
|  |  | rs2279686 | C/T | 0.49 | 0.38 |
| PARP1 | 1 | rs10915985 | C/T | 0.43 | 0.86 |
|  |  | rs6426551 | G/A | 0.25 | 1.00 |
|  |  | rs6677172 | C/G | 0.41 | 0.85 |
|  |  | rs12568297 | G/C | 0.30 | 0.16 |
|  |  | rs6668722 | T/C | 0.07 | 0.54 |
|  |  | rs12143101 | G/C | 0.17 | 0.64 |
|  |  | rs8679 | A/G | 0.29 | 0.47 |
|  |  | rs2271347 | G/A | 0.28 | 1.00 |
|  |  | rs752307 | G/C | 0.18 | 0.91 |
|  |  | rs3219142 | G/A | 0.15 | 0.06 |
|  |  | rs1136410 | A/G | 0.18 | 0.74 |
|  |  | rs3219123 | G/A | 0.09 | 0.70 |
|  |  | rs3219110 | T/C | 0.40 | 3.27×10^-4^ |
|  |  | rs1805401 | C/G | 0.04 | 1.40×10^-4^ |
|  |  | rs3219104 | C/A | 0.18 | 0.74 |
|  |  | rs3219095 | T/C | 0.14 | 0.06 |
|  |  | rs2271343 | C/G | 0.16 | 0.29 |
|  |  | rs2255403 | A/G | 0.18 | 0.57 |
|  |  | rs1002153 | T/C | 0.16 | 0.39 |
|  |  | rs7531668 | A/T | 0.16 | 0.18 |
|  |  | rs1104893 | A/G | 0.34 | 0.50 |
|  |  | rs2249844 | T/C | 0.34 | 0.95 |
|  |  | rs10915989 | G/A | 0.24 | 0.44 |
| PARP2 | 14 | rs7140961 | G/A | 0.14 | 0.24 |
|  |  | rs11622655 | A/G | 0.23 | 0.11 |
|  |  | rs7159947 | T/C | 0.38 | 0.70 |
|  |  | rs2297617 | T/C | 0.36 | 1.00 |
|  |  | rs3093882 | C/A | 0.22 | 0.54 |
|  |  | rs1760921 | T/C | 0.06 | 0.79 |
|  |  | rs3093904 | T/A | 0.22 | 0.44 |
|  |  | rs3093921 | A/G | 0.02 | 0.04 |
|  |  | rs3093926 | G/A | 0.10 | 0.86 |
|  |  | rs1760914 | A/G | 0.05 | 0.75 |
|  |  | rs3093930 | C/T | 0.35 | 0.75 |
|  |  | rs3093933 | G/T | 0.21 | 0.42 |
|  |  | rs878156 | T/C | 0.13 | 0.09 |
|  |  | rs2700 | A/C | 0.27 | 0.94 |
|  |  | rs3093942 | A/C | 0.20 | 0.63 |
|  |  | rs4981158 | T/C | 0.25 | 0.81 |
|  |  | rs10147163 | T/C | 0.25 | 1.00 |
|  |  | rs7161611 | C/A | 0.23 | 0.86 |
|  |  | rs1713419 | A/G | 0.48 | 0.02 |
| PIK3C3 | 18 | rs52911 | G/A | 0.37 | 1.00 |
|  |  | rs8087875 | A/G | 0.08 | 0.05 |
|  |  | rs504302 | A/G | 0.49 | 0.01 |
| PINX1 | 8 | rs718742 | T/G | 0.27 | 0.94 |
|  |  | rs10110552 | G/C | 0.09 | 7.18×10^-4^ |
|  |  | rs13258593 | G/A | 0.08 | 0.25 |
|  |  | rs17774023 | T/C | 0.26 | 0.28 |
|  |  | rs7826189 | G/A | 0.41 | 0.13 |
|  |  | rs11250076 | G/A | 0.44 | 0.41 |
|  |  | rs10094907 | T/A | 0.23 | 0.16 |
|  |  | rs10099928 | G/C | 0.23 | 0.32 |
|  |  | rs7002282 | G/A | 0.41 | 0.30 |
|  |  | rs6989930 | A/G | 0.09 | 0.20 |
|  |  | rs6995541 | A/G | 0.30 | 0.18 |
|  |  | rs6601530 | A/G | 0.49 | 0.45 |
|  |  | rs11779336 | C/T | 0.07 | 0.51 |
|  |  | rs6992039 | C/T | 0.24 | 0.34 |
|  |  | rs2409656 | G/C | 0.26 | 2.28×10^-3^ |
|  |  | rs13270324 | G/A | 0.04 | 0.64 |
|  |  | rs17152584 | G/A | 0.09 | 0.17 |
|  |  | rs6988598 | A/G | 0.33 | 0.10 |
|  |  | rs13259648 | G/T | 0.28 | 0.66 |
|  |  | rs6990589 | G/A | 0.16 | 0.92 |
|  |  | rs6989160 | C/A | 0.42 | 0.95 |
|  |  | rs1469557 | C/T | 0.16 | 0.84 |
|  |  | rs7012879 | G/C | 0.27 | 0.05 |
|  |  | rs10089153 | A/G | 0.32 | 0.84 |
|  |  | rs12545398 | T/C | 0.08 | 0.20 |
|  |  | rs17152619 | G/C | 0.09 | 0.08 |
|  |  | rs12546026 | A/C | 0.43 | 0.86 |
|  |  | rs877116 | T/G | 0.46 | 0.91 |
|  |  | rs2292369 | A/G | 0.43 | 0.76 |
| POT1 | 7 | rs17834698 | T/G | 0.04 | 0.14 |
|  |  | rs727505 | G/A | 0.30 | 0.30 |
|  |  | rs6973812 | C/T | 0.38 | 0.90 |
|  |  | rs4360236 | C/T | 0.09 | 0.87 |
|  |  | rs12539481 | G/T | 0.29 | 0.89 |
| PRKDC | 8 | rs4521758 | C/T | 0.12 | 0.75 |
|  |  | rs8178169 | T/C | 0.07 | 0.64 |
|  |  | rs8178071 | C/T | 0.24 | 0.61 |
|  |  | rs8178068 | C/T | 0.05 | 0.72 |
|  |  | rs2213178 | G/A | 0.34 | 0.74 |
| RAD50 | 5 | rs2299015 | T/G | 0.18 | 0.92 |
|  |  | rs10520117 | C/G | 0.01 | 1.00 |
|  |  | rs17622991 | G/A | 0.19 | 0.46 |
|  |  | rs10520114 | A/G | 0.18 | 0.57 |
|  |  | rs2301713 | T/C | 0.19 | 0.78 |
|  |  | rs17772565 | C/T | 0.07 | 0.67 |
|  |  | rs11955168 | C/T | 0.02 | 1.00 |
|  |  | rs12653750 | C/T | 0.18 | 0.85 |
|  |  | rs7737470 | T/A | 0.18 | 0.25 |
|  |  | rs2214370 | T/G | 0.01 | 0.17 |
|  |  | rs2158177 | A/G | 0.17 | 0.37 |
|  |  | rs739719 | C/A | 0.09 | 1.00 |
|  |  | rs2069812 | G/A | 0.33 | 0.95 |
|  |  | rs2522410 | C/T | 0.01 | 1.00 |
| RAD51AP1 | 12 | rs2970808 | C/T | 0.09 | 0.87 |
|  |  | rs10849083 | G/T | 0.10 | 0.62 |
|  |  | rs17701198 | G/A | 0.04 | 0.26 |
|  |  | rs7296957 | T/C | 0.12 | 0.77 |
|  |  | rs7965755 | G/T | 0.20 | 0.64 |
|  |  | rs740059 | G/A | 0.44 | 0.34 |
|  |  | rs7298545 | T/C | 0.27 | 0.17 |
|  |  | rs2884670 | G/A | 0.31 | 0.08 |
|  |  | rs7973740 | A/G | 0.09 | 0.72 |
|  |  | rs10450710 | T/C | 0.04 | 0.10 |
|  |  | rs10849094 | C/G | 0.05 | 0.75 |
|  |  | rs10444471 | G/T | 0.03 | 1.00 |
|  |  | rs10849096 | C/G | 0.40 | 0.46 |
| RAD51C | 17 | rs12939313 | A/T | 0.14 | 0.01 |
|  |  | rs302864 | G/A | 0.12 | 0.26 |
|  |  | rs6503874 | C/G | 0.06 | 0.07 |
|  |  | rs304268 | T/C | 0.45 | 0.16 |
|  |  | rs17822908 | T/C | 0.15 | 0.26 |
|  |  | rs304272 | C/A | 0.41 | 0.27 |
| RAD51L3 | 17 | rs9899949 | G/A | 0.02 | 0.17 |
|  |  | rs3785754 | G/T | 0.41 | 0.30 |
|  |  | rs4796033 | C/T | 0.13 | 0.54 |
|  |  | rs936656 | G/A | 0.45 | 0.22 |
|  |  | rs3816754 | G/T | 0.04 | 1.00 |
|  |  | rs8075760 | C/T | 0.41 | 0.55 |
|  |  | rs9915078 | A/G | 0.09 | 0.71 |
|  |  | rs1871892 | C/T | 0.29 | 0.79 |
|  |  | rs2306508 | C/A | 0.41 | 0.42 |
|  |  | rs17629188 | G/A | 0.18 | 0.84 |
|  |  | rs3736143 | C/T | 0.45 | 0.72 |
|  |  | rs10153277 | C/A | 0.02 | 0.53 |
|  |  | rs7215209 | T/C | 0.27 | 0.72 |
| RAD54L | 1 | rs6692775 | G/T | 0.14 | 0.73 |
|  |  | rs12141269 | C/T | 0.10 | 1.00 |
|  |  | rs10789488 | A/G | 0.18 | 0.12 |
|  |  | rs17102086 | T/C | 0.20 | 0.84 |
|  |  | rs12410307 | G/A | 0.14 | 0.03 |
|  |  | rs4660918 | T/C | 0.16 | 0.46 |
|  |  | rs17102098 | A/G | 0.02 | 0.11 |
|  |  | rs1048771 | C/T | 0.13 | 0.63 |
|  |  | rs12142240 | T/C | 0.27 | 0.66 |
| RECQL | 12 | rs10841820 | C/T | 0.04 | 0.27 |
|  |  | rs12424924 | G/A | 0.16 | 0.06 |
|  |  | rs10161132 | G/A | 0.37 | 0.85 |
|  |  | rs10492116 | C/T | 0.08 | 0.11 |
|  |  | rs1029931 | T/C | 0.19 | 0.92 |
|  |  | rs2284392 | G/A | 0.41 | 0.90 |
|  |  | rs11046081 | T/A | 0.08 | 0.04 |
|  |  | rs17627175 | T/G | 0.08 | 0.13 |
|  |  | rs11835261 | A/C | 0.09 | 0.14 |
|  |  | rs12829302 | C/T | 0.26 | 0.64 |
|  |  | rs2300211 | A/C | 0.48 | 0.64 |
|  |  | rs1061627 | A/G | 0.33 | 0.59 |
|  |  | rs1061626 | T/G | 0.22 | 0.21 |
|  |  | rs1860947 | A/C | 0.20 | 0.13 |
|  |  | rs11046087 | G/T | 0.08 | 0.71 |
|  |  | rs4762834 | C/T | 0.23 | 0.44 |
|  |  | rs10492117 | A/G | 0.41 | 0.76 |
|  |  | rs11046097 | C/T | 0.07 | 0.63 |
|  |  | rs2110162 | G/A | 0.07 | 1.00 |
|  |  | rs10841839 | A/G | 0.20 | 0.78 |
|  |  | rs3213213 | T/C | 0.21 | 0.59 |
| RECQL4 | 8 | rs756627 | G/A | 0.45 | 0.32 |
|  |  | rs4251689 | C/T | 0.47 | 0.16 |
|  |  | rs2721173 | T/C | 0.47 | 0.16 |
|  |  | rs10111332 | C/T | 0.44 | 0.91 |
|  |  | rs9071 | A/G | 0.48 | 0.03 |
| RECQL5 | 17 | rs820151 | G/A | 0.37 | 0.12 |
|  |  | rs820154 | A/T | 0.33 | 0.20 |
|  |  | rs9896073 | C/G | 0.28 | 0.33 |
|  |  | rs820199 | C/T | 0.33 | 0.36 |
| RTEL1 | 20 | rs2777933 | C/G | 0.02 | 1.00 |
|  |  | rs879471 | T/C | 0.44 | 0.17 |
|  |  | rs909334 | C/A | 0.20 | 0.41 |
|  |  | rs6089953 | G/A | 0.17 | 0.16 |
|  |  | rs2297434 | T/C | 0.49 | 0.73 |
|  |  | rs6011002 | A/G | 0.08 | 0.44 |
|  |  | rs2297437 | G/A | 0.23 | 0.67 |
|  |  | rs3787098 | C/T | 0.11 | 0.55 |
|  |  | rs6089956 | C/A | 0.08 | 1.00 |
|  |  | rs2738783 | G/T | 0.14 | 0.23 |
|  |  | rs4809324 | T/C | 0.11 | 0.36 |
|  |  | rs1291206 | G/A | 0.17 | 0.13 |
|  |  | rs6011040 | G/A | 0.26 | 0.14 |
| TEP1 | 14 | rs2104977 | G/A | 0.14 | 0.81 |
|  |  | rs7150689 | T/C | 0.28 | 0.82 |
|  |  | rs2297615 | A/T | 0.24 | 0.30 |
|  |  | rs938887 | A/G | 0.26 | 0.82 |
|  |  | rs1713455 | G/A | 0.14 | 0.43 |
|  |  | rs11850456 | G/A | 0.02 | 1.00 |
|  |  | rs12895477 | A/G | 0.13 | 0.65 |
|  |  | rs1713423 | G/A | 0.48 | 0.36 |
|  |  | rs1713417 | G/A | 0.08 | 0.54 |
|  |  | rs938892 | G/A | 0.08 | 0.85 |
|  |  | rs8009925 | C/A | 0.31 | 0.11 |
|  |  | rs2275009 | G/A | 0.10 | 0.08 |
|  |  | rs1760898 | G/T | 0.20 | 0.93 |
|  |  | rs3762145 | C/T | 0.13 | 4.08×10^-3^ |
|  |  | rs1713440 | T/A | 0.32 | 0.45 |
|  |  | rs1760897 | A/G | 0.28 | 0.62 |
|  |  | rs2678685 | G/T | 0.48 | 0.06 |
|  |  | rs2151753 | G/A | 0.12 | 0.06 |
|  |  | rs1760893 | A/C | 0.10 | 0.12 |
|  |  | rs1760891 | G/T | 0.15 | 1.00 |
|  |  | rs1713434 | T/C | 0.47 | 0.27 |
|  |  | rs1760890 | A/C | 0.11 | 0.87 |
|  |  | rs1760889 | G/T | 0.11 | 0.05 |
|  |  | rs12431912 | T/C | 0.35 | 0.69 |
|  |  | rs12436843 | G/A | 0.24 | 0.86 |
|  |  | rs6575809 | G/T | 0.44 | 0.27 |
|  |  | rs945012 | C/T | 0.38 | 0.09 |
|  |  | rs1953225 | T/C | 0.20 | 0.41 |
|  |  | rs17111534 | G/A | 0.20 | 0.65 |
|  |  | rs11160645 | C/T | 0.12 | 0.79 |
|  |  | rs11160653 | C/T | 0.23 | 0.10 |
| TERC | 3 | rs12638862 | A/G | 0.26 | 0.18 |
|  |  | rs2068178 | C/T | 0.02 | 1.00 |
|  |  | rs9860874 | C/A | 0.27 | 0.10 |
| TERF1 | 8 | rs1482029 | G/A | 0.43 | 0.95 |
|  |  | rs6994351 | A/G | 0.40 | 0.85 |
|  |  | rs6472718 | C/T | 0.17 | 0.24 |
|  |  | rs2929593 | C/T | 0.28 | 0.33 |
|  |  | rs3116136 | G/C | 0.23 | 0.74 |
|  |  | rs11993041 | C/T | 0.48 | 0.86 |
|  |  | rs9298211 | C/T | 0.28 | 0.42 |
|  |  | rs2929586 | A/G | 0.28 | 0.24 |
|  |  | rs10104094 | C/T | 0.48 | 0.77 |
|  |  | rs10095169 | T/C | 0.34 | 0.89 |
|  |  | rs10107605 | A/C | 0.15 | 0.08 |
|  |  | rs2306492 | G/A | 0.28 | 0.31 |
|  |  | rs1545827 | C/T | 0.48 | 0.91 |
|  |  | rs7001277 | G/A | 0.28 | 0.30 |
| TERF2 | 16 | rs16958777 | C/T | 0.04 | 0.33 |
|  |  | rs7191614 | A/G | 0.22 | 0.87 |
|  |  | rs7187579 | C/T | 0.16 | 0.67 |
|  |  | rs251796 | A/G | 0.30 | 0.23 |
|  |  | rs153045 | T/C | 0.34 | 0.80 |
|  |  | rs3785073 | G/A | 0.22 | 0.68 |
|  |  | rs9939705 | G/C | 0.22 | 0.13 |
| TERF2IP | 16 | rs7189895 | A/G | 0.31 | 0.23 |
|  |  | rs6564270 | C/T | 0.17 | 0.68 |
|  |  | rs11649388 | G/A | 0.17 | 0.61 |
|  |  | rs2289064 | C/A | 0.10 | 0.04 |
|  |  | rs8053257 | G/A | 0.07 | 1.00 |
|  |  | rs3784929 | A/G | 0.12 | 0.51 |
|  |  | rs11639771 | C/T | 0.14 | 0.23 |
| TERT | 5 | rs4075202 | C/A | 0.04 | 1.00 |
|  |  | rs4073918 | T/C | 0.23 | 0.36 |
|  |  | rs10078761 | T/A | 0.41 | 0.07 |
|  |  | rs2736122 | G/A | 0.22 | 0.80 |
|  |  | rs2736118 | T/C | 0.23 | 0.62 |
|  |  | rs4246742 | T/A | 0.13 | 0.19 |
|  |  | rs4975605 | C/A | 0.49 | 0.48 |
|  |  | rs10069690 | C/T | 0.25 | 0.94 |
|  |  | rs2242652 | G/A | 0.19 | 0.12 |
|  |  | rs2853676 | C/T | 0.30 | 0.18 |
|  |  | rs2735940 | A/G | 0.42 | 0.47 |
|  |  | rs2853668 | G/T | 0.32 | 0.54 |
| TINF2 | 14 | rs17794752 | G/C | 0.10 | 0.46 |
|  |  | rs3814815 | G/T | 0.07 | 1.00 |
|  |  | rs12891162 | A/C | 0.46 | 0.20 |
|  |  | rs7158744 | T/C | 0.32 | 1.00 |
|  |  | rs8193032 | A/C | 0.08 | 1.00 |
|  |  | rs3742506 | G/A | 0.07 | 0.66 |
|  |  | rs2748525 | G/A | 0.12 | 0.58 |
|  |  | rs7151201 | A/C | 0.17 | 0.84 |
|  |  | rs2748516 | G/A | 0.06 | 0.77 |
| TNKS | 8 | rs1471203 | T/C | 0.12 | 1.00 |
|  |  | rs7829065 | C/T | 0.08 | 0.82 |
|  |  | rs13261395 | G/C | 0.10 | 0.87 |
|  |  | rs11991621 | C/T | 0.15 | 0.56 |
|  |  | rs1458942 | A/G | 0.35 | 0.90 |
|  |  | rs11781665 | C/T | 0.10 | 0.38 |
|  |  | rs10503380 | C/T | 0.23 | 0.30 |
|  |  | rs6990097 | T/C | 0.26 | 0.17 |
|  |  | rs7834823 | C/A | 0.24 | 0.61 |
|  |  | rs12679892 | A/G | 0.29 | 0.31 |
|  |  | rs11787063 | G/T | 0.26 | 0.94 |
|  |  | rs6984737 | A/G | 0.08 | 0.84 |
|  |  | rs10090277 | A/G | 0.09 | 0.84 |
|  |  | rs10093972 | T/C | 0.09 | 0.85 |
|  |  | rs5002815 | G/T | 0.09 | 1.00 |
|  |  | rs5002814 | A/G | 0.09 | 0.85 |
|  |  | rs4474027 | A/G | 0.09 | 1.00 |
|  |  | rs13262577 | G/C | 0.24 | 0.08 |
|  |  | rs13250838 | C/T | 0.11 | 1.00 |
|  |  | rs7822904 | T/C | 0.23 | 0.31 |
|  |  | rs12679180 | A/G | 0.31 | 0.29 |
|  |  | rs9644704 | G/A | 0.35 | 0.95 |
|  |  | rs7825818 | G/T | 0.14 | 0.61 |
|  |  | rs7461376 | A/G | 0.25 | 0.41 |
|  |  | rs9644708 | T/C | 0.21 | 0.23 |
|  |  | rs4840437 | A/G | 0.36 | 0.19 |
|  |  | rs6995495 | C/G | 0.26 | 0.02 |
|  |  | rs11249943 | A/C | 0.17 | 0.52 |
|  |  | rs4289816 | T/C | 0.10 | 0.75 |
|  |  | rs12541709 | C/G | 0.21 | 0.86 |
|  |  | rs10100914 | C/A | 0.16 | 0.22 |
|  |  | rs17734024 | G/A | 0.08 | 0.16 |
|  |  | rs1055328 | C/G | 0.24 | 1.00 |
|  |  | rs17150478 | A/G | 0.17 | 0.52 |
|  |  | rs11787443 | C/T | 0.09 | 0.71 |
|  |  | rs7814160 | G/A | 0.16 | 0.91 |
|  |  | rs2898223 | C/T | 0.08 | 0.54 |
| TNKS2 | 10 | rs2421698 | C/T | 0.12 | 0.65 |
|  |  | rs1935031 | A/T | 0.39 | 0.72 |
|  |  | rs12411706 | G/A | 0.20 | 0.52 |
|  |  | rs2258946 | G/A | 0.16 | 0.13 |
|  |  | rs1772189 | T/A | 0.49 | 0.45 |
| WRN | 8 | rs7827210 | G/A | 0.39 | 0.18 |
|  |  | rs13278931 | G/T | 0.45 | 0.41 |
|  |  | rs2725335 | G/A | 0.06 | 1.00 |
|  |  | rs13269094 | T/G | 0.13 | 0.04 |
|  |  | rs2737315 | C/T | 0.20 | 0.85 |
|  |  | rs2015230 | T/C | 0.34 | 0.95 |
|  |  | rs11574212 | A/T | 0.02 | 0.09 |
|  |  | rs2725385 | C/T | 0.30 | 0.89 |
|  |  | rs1800391 | G/A | 0.09 | 0.46 |
|  |  | rs13251813 | C/T | 0.04 | 0.06 |
|  |  | rs4733225 | T/C | 0.20 | 1.51×10^-3^ |
|  |  | rs3024239 | T/C | 0.48 | 0.81 |
|  |  | rs6982140 | T/C | 0.06 | 1.00 |
|  |  | rs1346044 | T/C | 0.24 | 0.80 |
|  |  | rs919002 | T/C | 0.39 | 0.76 |
|  |  | rs16877804 | T/C | 0.08 | 0.68 |
| XRCC6 | 22 | rs80477 | T/C | 0.24 | 0.74 |
|  |  | rs132767 | C/T | 0.22 | 0.16 |
|  |  | rs2267437 | C/G | 0.40 | 0.23 |
|  |  | rs11703638 | G/A | 0.30 | 0.26 |
