# Supplementary material for: On the Interplay of Telomeres, Nevi and the Risk of Melanoma
Source: PLoS One. 2012 Dec 27;7(12):e52466. doi: 10.1371/journal.pone.0052466 (PMC3531488; doi:10.1371/journal.pone.0052466)
Supplement: Table S2 — (DOC) [file pone.0052466.s010.doc]

**Table S2.** SNP-based association analysis between the SNPs in the RECQL4 region and the risk of melanoma.

| SNP | OR^*^ | (95% CI) | P-trend | MAF^§^ | OR^**^ | (95% CI)^**^ | P-trend^**^ |
| --- | --- | --- | --- | --- | --- | --- | --- |
| rs2721173 | 1.35 | (1.13, 1.62) | 1.13×10^-3^ | 0.47 | 1.56 | (1.12, 2.18) | 8.58×10^-3^ |
| rs9071 | 1.29 | (1.07, 1.55) | 7.43×10^-3^ | 0.48 | 1.42 | (1.14, 1.78) | 1.96×10^-3^ |
| rs756627 | 1.27 | (1.07, 1.52) | 7.44×10^-3^ | 0.45 | 1.45 | (1.10, 1.91) | 7.71×10^-3^ |
| rs4251689 | 1.30 | (1.06, 1.60) | 0.01 | 0.47 | 1.52 | (1.09, 2.13) | 0.01 |
| rs10111332 | 1.12 | (0.94, 1.34) | 0.22 | 0.44 | 1.23 | (1.00, 1.51) | 0.05 |

^*^Adjusted by age and sex. ^§^In subjects without melanoma.

^**^Adjusted by age, sex and factor 2 (related to presence of dysplastic nevi and nevi count).
