# Supplementary material for: On the Interplay of Telomeres, Nevi and the Risk of Melanoma
Source: PLoS One. 2012 Dec 27;7(12):e52466. doi: 10.1371/journal.pone.0052466 (PMC3531488; doi:10.1371/journal.pone.0052466)
Supplement: Table S3 — (DOC) [file pone.0052466.s011.doc]

**Table S3.** Association analysis between rs2721173 in the RECQL4 region and the risk of melanoma by study.

| Study | OR^*^ | (95% CI) | P-trend |
| --- | --- | --- | --- |
| CCS1 | 1.06 | (0.74-1.53) | 0.75 |
| CCS2 | 1.46 | (1.07-2.00) | 0.02 |
| CCS3 | 1.43 | (1.04-1.96) | 0.03 |
| FS | 1.72 | (0.90-3.30) | 0.10 |
| Overall | 1.35 | (1.13-1.62) | 1.13×10^-3^ |

^*^Adjusted by age and sex.

Quantifying heterogeneity: I^2^=0% [0%, 82.1%]

Test of heterogeneity: Q=2.57, P-value=0.46.
