# Supplementary material for: On the Interplay of Telomeres, Nevi and the Risk of Melanoma
Source: PLoS One. 2012 Dec 27;7(12):e52466. doi: 10.1371/journal.pone.0052466 (PMC3531488; doi:10.1371/journal.pone.0052466)
Supplement: Table S4 — (DOC) [file pone.0052466.s012.doc]

**Table S4.** SNP-based association analysis with the risk of dysplastic nevi for SNPs in the RTEL1 region in non-melanoma subjects.

| SNP | OR^*^ | (95% CI) | P-trend | MAF^§^ |
| --- | --- | --- | --- | --- |
| rs6011002 | 3.30 | (1.64, 6.61) | 7.75×10^-4^ | 0.08 |
| rs6011040 | 1.56 | (0.91, 2.67) | 0.10 | 0.26 |
| rs909334 | 0.82 | (0.46, 1.47) | 0.51 | 0.20 |
| rs3787098 | 0.86 | (0.42, 1.75) | 0.67 | 0.11 |
| rs4809324 | 0.88 | (0.41, 1.89) | 0.75 | 0.11 |
| rs2297434 | 1.13 | (0.51, 2.48) | 0.77 | 0.49 |
| rs6089956 | 1.12 | (0.49, 2.58) | 0.79 | 0.08 |
| rs2738783 | 0.93 | (0.50, 1.74) | 0.83 | 0.14 |
| rs879471 | 1.09 | (0.48, 2.50) | 0.83 | 0.44 |
| rs1291206 | 0.95 | (0.52, 1.73) | 0.86 | 0.17 |
| rs2297437 | 0.95 | (0.54, 1.68) | 0.87 | 0.23 |
| rs6089953 | 0.97 | (0.44, 2.10) | 0.93 | 0.17 |

^*^Adjusted by age and sex. ^§^In subjects without dysplastic nevi nor melanoma.
