# Supplementary material for: On the Interplay of Telomeres, Nevi and the Risk of Melanoma
Source: PLoS One. 2012 Dec 27;7(12):e52466. doi: 10.1371/journal.pone.0052466 (PMC3531488; doi:10.1371/journal.pone.0052466)
Supplement: Table S5 — (DOC) [file pone.0052466.s013.doc]

**Table S5.** Association analysis between rs6011002 in the RTEL1 region and the risk of dysplastic nevi in non-melanoma subjeccts by study.

| Study | OR^*^ | (95% CI) | P-trend |
| --- | --- | --- | --- |
| CCS1 | 3.77 | (1.30, 10.93) | 0.01 |
| CCS2 | 1.82 | (0.43, 7.80) | 0.41 |
| CCS3 | 3.73 | (0.92, 15.05) | 0.06 |
| FS | 5.49 | (0.56, 53.43) | 0.14 |
| Overall | 3.30 | (1.64, 6.61) | 7.75×10^-4^ |

^*^Adjusted by age and sex.

Quantifying heterogeneity: I^2^=0% [0%, 50%]

Test of heterogeneity: Q=0.92, P-value=0.82.
