# Supplementary material for: On the Interplay of Telomeres, Nevi and the Risk of Melanoma
Source: PLoS One. 2012 Dec 27;7(12):e52466. doi: 10.1371/journal.pone.0052466 (PMC3531488; doi:10.1371/journal.pone.0052466)
Supplement: Table S6 — (DOC) [file pone.0052466.s014.doc]

**Table S6.** Association analysis between rs11955168 in the RAD50 region and nevus count by study.

| Study | IRR^*^ | (95% CI) | P-trend |
| --- | --- | --- | --- |
| CCS1 | 2.25 | (1.09, 4.64) | 0.03 |
| FS | 3.36 | (1.74, 6.51) | 3.10×10^-4^ |
| Overall | 2.79 | (1.77, 4.38) | 8.68×10^-6^ |

^*^Adjusted by age, sex and an interaction term of age and nevus count.

Quantifying heterogeneity: I^2^=0%

Test of heterogeneity: Q=0.65, P-value=0.42.
