# Supplementary material for: On the Interplay of Telomeres, Nevi and the Risk of Melanoma
Source: PLoS One. 2012 Dec 27;7(12):e52466. doi: 10.1371/journal.pone.0052466 (PMC3531488; doi:10.1371/journal.pone.0052466)
Supplement: Table S7 — (DOC) [file pone.0052466.s015.doc]

**Table S7.** Association analysis between rs11850456 in the TEP1 region and nevus count by study.

| Study | IRR^*^ | (95% CI) | P-trend |
| --- | --- | --- | --- |
| CCS1 | 0.39 | (0.20, 0.77) | 6.45×10^-3^ |
| FS | 0.64 | (0.17, 2.35) | 0.50 |
| Overall | 0.41 | (0.26, 0.64) | 1.04×10^-4^ |

^*^Adjusted by age, sex and an interaction term of age and nevus count.

Quantifying heterogeneity: I^2^=0%

Test of heterogeneity: Q=0.43, P-value=0.51.
