# Supplementary material for: On the Interplay of Telomeres, Nevi and the Risk of Melanoma
Source: PLoS One. 2012 Dec 27;7(12):e52466. doi: 10.1371/journal.pone.0052466 (PMC3531488; doi:10.1371/journal.pone.0052466)
Supplement: Table S8 — (DOC) [file pone.0052466.s016.doc]

**Table S8.** SNP-based association analysis with nevus count for SNPs in the TERF2 region among non-melanoma subjects.

| SNP | IRR^*^ | (95% CI) | P-trend | MAF^§^ |
| --- | --- | --- | --- | --- |
| rs153045 | 1.49 | (1.20, 1.85) | 3.27×10^-4^ | 0.34 |
| rs16958777 | 0.41 | (0.23, 0.73) | 2.30×10^-3^ | 0.04 |
| rs251796 | 1.32 | (1.06, 1.66) | 0.01 | 0.30 |
| rs9939705 | 0.85 | (0.64, 1.14) | 0.28 | 0.22 |
| rs3785073 | 0.88 | (0.67, 1.16) | 0.36 | 0.22 |
| rs7191614 | 0.91 | (0.69, 1.19) | 0.47 | 0.22 |
| rs7187579 | 1.11 | (0.83, 1.48) | 0.50 | 0.16 |

^*^Adjusted by age, sex and an interaction of age and nevus count. ^§^In subjects without dysplastic nevi nor melanoma.
