# Supplementary material for: On the Interplay of Telomeres, Nevi and the Risk of Melanoma
Source: PLoS One. 2012 Dec 27;7(12):e52466. doi: 10.1371/journal.pone.0052466 (PMC3531488; doi:10.1371/journal.pone.0052466)
Supplement: Table S9 — (DOC) [file pone.0052466.s017.doc]

**Table S9.** Association analysis between rs153045 in the TERF2 region and nevus count by study.

| Study | IRR^*^ | (95% CI) | P-value |
| --- | --- | --- | --- |
| CCS1 | 1.26 | (0.85, 1.85) | 0.25 |
| FS | 1.71 | (1.08, 2.72) | 0.02 |
| Overall | 1.49 | (1.20, 1.85) | 3.27×10^-4^ |

^*^Adjusted by age, sex and an interaction term of age and nevus count.

Quantifying heterogeneity: I^2^=0%

Test of heterogeneity: Q=1.01, P-value=0.32.
