# Supplementary material for: On the Interplay of Telomeres, Nevi and the Risk of Melanoma
Source: PLoS One. 2012 Dec 27;7(12):e52466. doi: 10.1371/journal.pone.0052466 (PMC3531488; doi:10.1371/journal.pone.0052466)
Supplement: Table S10 — (DOC) [file pone.0052466.s018.doc]

**Table S10.** Association analysis between rs rs251796 in the TERF2 region and nevus count by study.

| Study | IRR^*^ | (95% CI) | P-value |
| --- | --- | --- | --- |
| CCS1 | 1.14 | (0.80, 1.64) | 0.47 |
| FS | 1.34 | (0.70, 2.55) | 0.38 |
| Overall | 1.32 | (1.06, 1.66) | 0.01 |

^*^Adjusted by age, sex and an interaction term of age and nevus count.

Quantifying heterogeneity: I^2^=0%

Test of heterogeneity: Q=0.18, P-value=0.67.
