# Supplementary material for: On the Interplay of Telomeres, Nevi and the Risk of Melanoma
Source: PLoS One. 2012 Dec 27;7(12):e52466. doi: 10.1371/journal.pone.0052466 (PMC3531488; doi:10.1371/journal.pone.0052466)
Supplement: Table S11 — (DOC) [file pone.0052466.s019.doc]

**Table S11.** Association between telomere-associated functional groups and risk of melanoma, dysplastic nevi and nevus count.

| Functional group^§^ | Genes in functional group | Melanoma  P-value^*^ | Dysplastic nevi  P-value^**^ | Nevus count  P-value^***^ |
| --- | --- | --- | --- | --- |
| Telomerase associated | NOLA2, NOLA3, TEP1, TERC, TERT | 0.76 | 0.82 | 0.13 |
| Shelterin | ACD, POT1, TERF1, TERF2, TERF2IP, TINF2 | 0.75 | 1.00 | 0.09 |
| Other telomere | MEN1, MYC, NOLA1, PARP1, PARP2, PIK3C3, PINX1, RTEL1, TNKS, TNKS2 | 0.67 | 0.11 | 0.34 |
| Helicase | BLM, DDX1, DDX11, RECQL, RECQL4, RECQL5, WRN | **0.03** | 0.79 | 0.12 |
| DNA repair | ATM, MCM4, MRE11A, NBN, RAD50, RAD51AP1, RAD51C, RAD51L3, RAD54L, XRCC6 | 0.73 | 0.27 | 0.77 |

^§^As defined in Mirabello *et al.* (2011).

^*^ Based on a meta-analysis of three case-control studies and a family study. Model was adjusted for age (continous) and sex.

^**^ In non-melanoma subjects. Based on a meta-analysis of three case-control studies and a family study. Model was adjusted for age (continous) and sex.

^***^ In non-melanoma subjects. Based on a meta-analysis of one case-control study and a family study using Poissson regression with robust variance. Model was adjusted for age (continous) and sex and interaction between age and SNP genotype.
